# Thyroid Hormone T4 Alleviates Traumatic Brain Injury by Enhancing Blood–Brain Barrier Integrity

**DOI:** 10.3390/ijms26199632

**Published:** 2025-10-03

**Authors:** Mayuri Khandelwal, Zhe Ying, Fernando Gomez-Pinilla

**Affiliations:** 1 Department of Integrative Biology and Physiology, University of California Los Angeles, Los Angeles, CA 90095, USA; 2 Department of Neurosurgery, University of California Los Angeles, Los Angeles, CA 90095, USA

**Keywords:** blood–brain barrier, traumatic brain injury, thyroid hormone, cognition

## Abstract

Traumatic brain injury (TBI) disrupts the blood–brain barrier (BBB), resulting in increased permeability, neuronal loss, and cognitive dysfunction. This study investigates the therapeutic potential of thyroid hormone (T4) to reduce BBB dysfunction following moderate fluid percussion injury. T4 injection (intraperitoneal) after TBI restores the levels of pericytes and endothelial cells vital for BBB integrity, reduces edema by downregulating AQP-4 gene expression, and enhances levels of the tight junction protein ZO-1. T4 counteracts the TBI-related increase in MMP-9 and TLR-4, significantly reducing BBB permeability. Furthermore, T4 enhances the neuroprotective functions of astrocytes by promoting the activity of A2 astrocytes. Additionally, T4 treatment increases DHA levels (important for membrane integrity and function), stimulates mitochondrial biogenesis, and leads to a notable improvement in spatial learning and memory retention. These findings suggest that T4 has significant potential to reduce vascular leakage and inflammation after TBI, thereby improving cognitive function and maintaining BBB integrity.

## 1. Introduction

The blood–brain barrier (BBB) shields the brain from chemicals transported in blood and plays an important role in the clearance of toxins [1,2,3] to maintain brain homeostasis [4]. The selective exchange of nutrients and metabolites between brain and blood can occur through simple diffusion, receptor-mediated transcytosis, or carrier-mediated transport [5]. BBB is composed of a neurovascular unit (NVU) that comprises endothelial cells connected by tight junctions [6,7], pericytes, astrocytes, and other cerebral cell types. As a consequence of traumatic brain injury (TBI), blood vessels are damaged and tight junctions are disrupted resulting in increased paracellular permeability, neuronal loss, and cognitive dysfunction [8]. Astrocytes surrounding the micro-vascular wall of BBB form an intricate network with endothelial cells interacting in a paracrine manner critical for the maintenance of BBB integrity [9,10]. Extensive damage to BBB causes brain edema leading to ischemia [11] and infiltration of inflammatory cytokines into the traumatized brain parenchyma [8,12].

The platelet-derived growth factor (PDGF) family encompasses four distinct ligands, denoted as PDGF-A through PDGF-D. Among these, the interaction between PDGF-B and its receptor (PDGFRβ) holds particular significance for orchestrating the recruitment of pericytes, pivotal for maintaining the integrity of the BBB [13]. PDGF-B is secreted by endothelial cells and binds to PDGFRβ on pericytes for maintaining permeability and tightness of the NVU by activating downstream signaling. However, injury triggers the binding of PDGF-B to PDGFRβ and increases the permeability of toxins across the BBB.

Thyroid hormone is crucial for brain development and functions. Hypothyroidism is a complication found in individuals suffering from TBI causing neuronal impairment and intellectual deficit [14]. Membrane transporters facilitate the transport of thyroid hormone T4 and prohormone T3 across the BBB. Thyroid hormone exerts its effects by binding to its receptors THα1 and THβ1. THα1 is more responsive to T4 while THβ1 to T3. In brain, the abundance of THα1 is higher in comparison to THβ1. The main thyroid transporters found in brain are monocarboxylate transporter 8 (MCT8) and the organic anion transporter polypeptide 1C1 (OATP1C1). MCT8 predominantly facilitates the transport of T4 and T3 from endothelial cells of the BBB to astrocytes. On the other hand, OATP1C1 primarily transports T4 from astrocytes to neurons, finely regulating thyroid hormone function within neural networks [15]. The objective of this study is to further elucidate the action of thyroid hormone on BBB integrity and permeability.

## 2. Results

### 2.1. Effect of T4 on Vascular Leakage in Frontal Cortex and Hippocampus 7 Days Post-TBI

**Figure 1:**
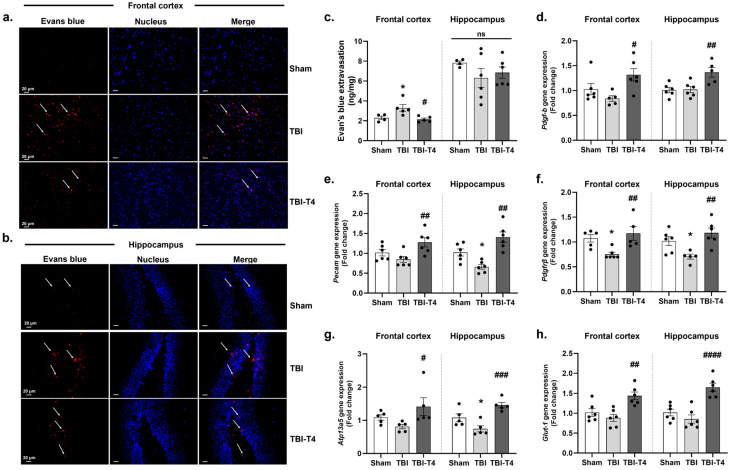
T4 ameliorated BBB leakage by regulating pericytes and endothelial cells 7 days post-TBI. Evan’s blue staining for BBB leakage in (**a**) frontal cortex, (**b**) hippocampus, (**c**) quantification in frontal cortex and hippocampus. Gene expression in frontal cortex and hippocampus: (**d**) PDGF-B (endothelial marker), (**e**) PECAM (CD-31), an endothelial marker, (**f**) PDGFRβ, (**g**) ATP13a5, (**h**) glucose transporter 1 (GLUT-1). Data are expressed as mean ± SEM, *n* = 4–6 mice/group. * *p* < 0.05 (sham vs. TBI). **^#^** *p* < 0.05, **^##^** *p* < 0.01, **^###^** *p* < 0.001, **^####^** *p* < 0.0001 (TBI vs. TBI-T4), ns represents non-significant. Data was analyzed using ANOVA followed by post hoc test.

Qualitatively, our results showed that Evan’s blue dye signal was stronger in the frontal cortex in the TBI group as compared to sham, and T4 administration counteracted this increase in response to TBI (Figure 1a), while no difference was found in the hippocampus (Figure 1b). For quantification of Evan’s blue, extravasation was measured in the frontal cortex and hippocampus isolated from dye-injected mice. One-way ANOVA analysis of Evan’s blue in brain tissue showed F (2, 11) = 7.03, *p* = 0.0108, in the frontal cortex, and F (2, 13) = 0.98, *p* = 0.4006, in the hippocampus, followed by Sidak’s post hoc test for the frontal cortex. In the frontal cortex, TBI showed larger internalization of dye compared to sham (*p* = 0.0304), while no significant changes in Evan’s blue uptake were detected in the hippocampus. T4 treatment resulted in reduced entry of dye in the frontal cortex (*p* = 0.0102); however, no significant differences were observed in the hippocampus among the groups (Figure 1c). To obtain a phenotypic account, we performed immunohistochemistry for Evan’s blue staining. We observed extravasation in approximation to the nuclei and cell body in the frontal cortex. Mice with TBI showed Evan’s blue internalization in the frontal cortex region of the brain with restricted entry in the hippocampus probably due to the presence of physical barriers. Evan’s blue with DAPI was particularly useful for easy visualization of the cell under a microscope. We proceed further to assess the BBB permeability by monitoring pericyte and endothelial cell disruption and the effects of other BBB markers both on the frontal cortex and hippocampus.

PDGF-B is an endothelial marker that, upon secretion, binds to pericytes. One-way ANOVA analysis of *Pdgf-b* showed F (2, 14) = 4.94, *p* = 0.0238, in the frontal cortex and F (2, 14) = 8.01, *p* = 0.0048, in the hippocampus, followed by Sidak’s post hoc test. Although not statistically significant, data showed possible effects of reduced gene expression of endothelial marker *Pdgf-b*, after TBI, compared to sham in the frontal cortex but not in the hippocampus. However, T4 treatment increased the gene expression of *Pdgf-b* in the frontal cortex (*p* = 0.0158) and hippocampus (*p* = 0.0076) (Figure 1d). One-way ANOVA analysis of *Pecam* (endothelial marker) showed F (2, 15) = 5.52, *p* = 0.0159, in the frontal cortex and F (2, 15) = 15.04, *p* = 0.0003, in the hippocampus followed by Sidak’s post hoc test. Gene expression of *Pecam* showed a reduction in the TBI group compared to the sham group in the hippocampus (*p* = 0.0352) and no significant difference in the frontal cortex. T4 administration counteracted the effects of TBI on *Pecam* both in the frontal cortex (*p* = 0.0097) and hippocampus (*p* = 0.0001) in response to the TBI group (Figure 1e).

Pericytes are indispensable elements of the BBB and play a dynamic role in regulating unwanted traffic of substances across the BBB and preserving neurovascular functions. *Pdgfrβ* is the most prominent and well-known molecular marker for pericytes. *Pdgfrβ* showed brain-region-specific changes affecting the frontal cortex and hippocampus post-TBI. One-way ANOVA analysis of *Pdgfrβ* showed F (2, 13) = 6.39, *p* = 0.0117, in the frontal cortex and F (2, 14) = 7.38, *p* = 0.0065, in the hippocampus followed by Sidak’s post hoc test. *Pdgfrβ* was found to be reduced in the 7-days-post-TBI group compared to sham group (frontal cortex, *p* = 0.0449; hippocampus, *p* = 0.047), while T4 treatment counteracted the reducing effects of TBI on the mRNA levels of *Pdgfrβ* (T4 vs. TBI, frontal cortex *p* = 0.0099; hippocampus, *p* = 0.0038) (Figure 1f). Apart from the various well-established pericyte markers, *Atp13a5* is highly specific to CNS while other known markers such as *Pdgfrβ* are expressed in other tissues as well, i.e., liver, kidney, etc. [16]. One-way ANOVA analysis for *Atp13a5* showed F (2, 12) = 3.36, *p* = 0.0152, in the frontal cortex and F (2, 12) = 13.05, *p* = 0.0010 in the hippocampus followed by Sidak’s post hoc test. In the hippocampus, TBI reduced the expression of *Atp13a5* in comparison to sham (*p* = 0.0456), while in the frontal cortex there was a reduction trend. However, T4 administration sharply increased the mRNA levels of *Atp13a5* counteracting the reduced levels in the frontal cortex (T4 vs. TBI, *p* = 0.0466) and hippocampus of TBI animals (T4 vs. TBI, *p* = 0.0005) (Figure 1g).

Glucose transporters (GLUT) play a crucial role in ensuring a constant supply of glucose to meet the high energy demands of the brain. Among these transporters, GLUT1 stands out as predominantly located by the BBB, emphasizing its vital role in facilitating glucose transport into the brain. Moreover, GLUT1 plays an important role in the development of the BBB because of its high glucose utilization and energy demands. One-way ANOVA analysis of *Glut-1* showed F (2, 15) = 9.44, *p* = 0.0022, in the frontal cortex and F (2, 15) = 18.68, *p* < 0.0001, in the hippocampus. Sidak’s post hoc test showed that T4 treatment increased the expression of *Glut-1* after TBI both in the frontal cortex and hippocampus in comparison to TBI (frontal cortex; *p* = 0.0017, hippocampus; *p* = 0.0001) (Figure 1h).

### 2.2. Effect of T4 on Thyroid Hormone Receptor and Transporters

**Figure 2:**
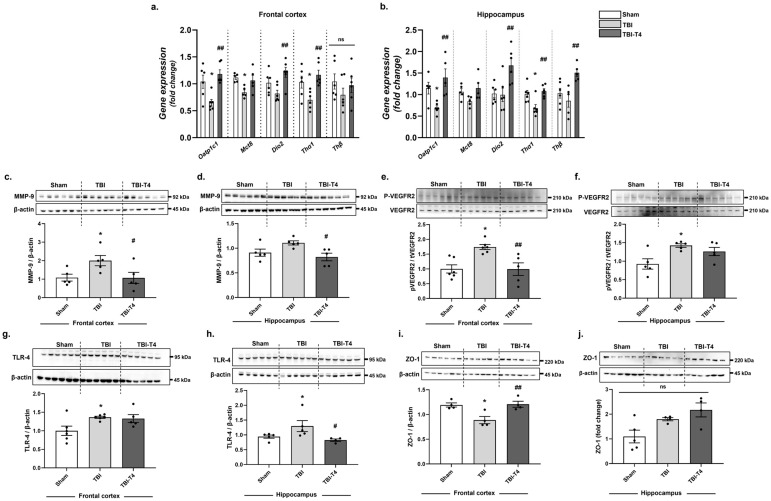
T4 regulates hyperpermeability and gene expression of its transporters with BBB leakage and protects the tight junction 7 days post-TBI both in frontal cortex and hippocampus. Gene expression of OATP1, MCT8, *Dio2*, THα1, THβ (**a**) in frontal cortex (**b**) in hippocampus. T4 ameliorated hyperpermeability 7 days post TBI; protein levels of MMP-9 normalized with β-actin (**c**) in frontal cortex; (**d**) in hippocampus. Protein levels of pVEGFR2 normalized with total VEGFR2 (**e**) in frontal cortex; (**f**) in hippocampus. Protein levels of TLR-4 normalized with β-actin (**g**) in frontal cortex; (**h**) in hippocampus. Protein levels of tight junction proteins, ZO-1 normalized with β-actin (**i**) in frontal cortex; (**j**) in hippocampus. Data are expressed as mean ± SEM, *n* = 4–6 mice/group. * *p* < 0.05 (sham vs. TBI). **^#^** *p* < 0.05, **^##^** *p* < 0.01 (TBI vs. TBI-T4), ns represents non-significant. Data was analyzed using ANOVA followed by post hoc test.

Organic anion transporter polypeptide 1C1 (OATP1C1) and monocarboxylate transporter 8 (MCT8) are the integral components of the BBB and responsible for facilitating the transport of TH into the brain. One-way ANOVA analysis of *Oatp1c1* showed F (2, 15) = 9.17, *p* = 0.0025, in the frontal cortex and F (2, 13) = 7.44, *p* = 0.0070, in the hippocampus followed by Sidak’s post hoc test. Our results showed that in the frontal cortex, the mRNA levels of *Oatp1c1* are reduced post-TBI compared to sham (*p* = 0.0166), and these effects were counteracted by T4 administration (T4 vs. TBI, *p* = 0.0018). TBI reduced TH transporter *Oatp1c1* mRNA in the hippocampus compared to sham (*p* = 0.0489), while T4 treatment reinstated the gene levels (*p* = 0.0047) (Figure 2a,b).

Mutation in MCT8 in humans results in impaired neurodevelopmental effects, along with reduced circulating TH levels [17,18]. One-way ANOVA analysis of *Mct8* showed F (2, 12) = 5.03, *p* = 0.0259, in the frontal cortex and F (2, 12) = 3.10, *p* = 0.0818 in the hippocampus followed by Sidak’s post hoc test for the frontal cortex. Our results showed that TBI decreased levels of *Mct8* mRNA transporter in the frontal cortex (*p* = 0.0220) and had a decreasing trend in the hippocampus, as compared to sham. Although not statistically significant, a decreasing possibility in the hippocampus was observed, as compared to sham. However, T4 administration in the frontal cortex had an increasing possibility, though non-significant in Mct8 mRNA as compared to the TBI group (Figure 2a,b).

We also assessed the mRNA levels of *Dio2*, an enzyme important for the conversion of T4 to T3 by removing 5′iodine from T4. One-way ANOVA analysis of *Dio2* showed F (2, 15) = 6.06, *p* = 0.0117, in the frontal cortex and F (2, 15) = 7.51, *p* = 0.0055, in the hippocampus followed by Sidak’s post hoc test. Although TBI did not affect the mRNA levels of *Dio2* in the frontal cortex and hippocampus, T4 treatment increased these levels in TBI animals that could translate into higher conversion of T4 to T3 as compared to the TBI group (frontal cortex, *p* = 0.0067; hippocampus, *p* = 0.0075) (Figure 2a,b).

Thyroid hormone receptor THRα is abundant in the brain and helps in the transport of T4 via receptor binding. One-way ANOVA analysis of *Thα1* showed F (2, 15) = 6.84, *p* = 0.0077, in the frontal cortex and F (2, 15) = 8.11, *p* = 0.0041, in the hippocampus followed by Sidak’s post hoc test. Our results showed that mRNA levels of *Thα1* were drastically reduced in TBI mice as compared to sham (frontal cortex, *p* = 0.0439; hippocampus, *p* = 0.0135) while T4 administration counteracted these reductions (frontal cortex, *p* = 0.0053; hippocampus, *p* = 0.0038) (Figure 2a,b). One-way ANOVA analysis of *Thβ1* showed F (2, 12) = 2.66, *p* = 0.1103, in the frontal cortex and F (2, 12) = 7.88, *p* = 0.0065, in the hippocampus followed by Sidak’s post hoc test for the hippocampus. *Thβ1* mRNA levels were not affected by TBI in comparison to sham. However, T4 administration in TBI mice increased mRNA levels of *Thβ1* in hippocampus as compared to TBI group (hippocampus, *p* = 0.0040) (Figure 2a,b).

### 2.3. Effect of T4 on Hyperpermeability and Angiogenesis Post-TBI

BBB leakage results in brain edema after injury and is associated with substantial increases in matrix metalloproteins (MMPs). Among the various MMPs, MMP-9 is the most prominent and majorly affected post injury [19]. One-way ANOVA analysis of MMP-9 showed F (2, 12) = 4.26, *p* = 0.040, in the frontal cortex and F (2, 12) = 4.94, *p* = 0.0271 in the hippocampus followed by Sidak’s post hoc test. The protein level of MMP-9 increased in the frontal cortex of TBI animals (*p* = 0.0440), while a non-significant increase was observed in the hippocampus in comparison to sham (*p* = 0.1060). However, T4 treatment counteracted the increase observed after TBI (T4 vs. TBI; frontal cortex, *p* = 0.0403; hippocampus, *p* = 0.0193) (Figure 2c,d).

MMP-9 mediates BBB hyperpermeability working on the toll-like receptor 4 (TLR4), and level of MMP-9 was increased in the traumatized brain. One-way ANOVA analysis of TLR-4 showed F (2, 14) = 4.58, *p* = 0.0294, in the frontal cortex and F (2, 11) = 4.046, *p* = 0.0482, in the hippocampus followed by Sidak’s post hoc test. In conjunction with MMP-9, TLR-4 was also increased in TBI mice vs. sham both in the frontal cortex and hippocampus (frontal cortex, *p* = 0.029, hippocampus, *p* = 0.0319). However, T4 treatment counteracted the TBI-related increase in TLR-4 levels in the hippocampus but not in the frontal cortex (frontal cortex, *p* = 0.66, hippocampus, *p* = 0.0433) (Figure 2g,h).

Astrocytes play a crucial role in preserving the integrity of the BBB through the secretion of vital factors like VEGF that supports angiogenesis and vascular permeability. However, in conditions such as TBI or stroke, elevations of VEGF can have detrimental effects on BBB integrity. One-way ANOVA analysis of VEGFR2 showed F (2, 14) = 8.79, *p* = 0.0034, in the frontal cortex and F (2, 12) = 5.64, *p* = 0.0187, in the hippocampus followed by Sidak’s post hoc test. Our findings indicate that 7 days post-TBI, the phosphorylation of VEGFR2 was increased both in the frontal cortex and hippocampus in comparison to sham (TBI vs. sham; frontal cortex, *p* = 0.0050, hippocampus, *p* = 0.0128). However, T4 administration reinstated the levels of pVEGFR2 in the frontal cortex (*p* = 0.0060) while a non-significant reduction was observed in hippocampus compared to TBI (Figure 2e,f). Upregulation of VEGF in TBI or stroke damages the BBB by activating MMP-9 while its inhibition provides protection [20]. Accordingly, we found VEGF is expressed in endothelial cells, astrocytes and neurons after cerebral injury and exerts an action on angiogenesis [21]. ZO-1 is an important tight junction protein that helps filter toxins and maintains the integrity of the blood–brain barrier. One-way ANOVA analysis of ZO-1 showed F (2, 9) = 9.05, *p* = 0.0070, in the frontal cortex and F (2, 10) = 5.79, *p* = 0.0213, in the hippocampus followed by Sidak’s post hoc test. TBI also resulted in reduction in tight junction protein ZO-1 relative to sham (*p* = 0.0115) while the effects were counteracted by T4 treatment in the frontal cortex (*p* = 0.0088) (Figure 2i). However, no significant changes were observed in the hippocampus (Figure 2j).

### 2.4. Post-TBI Effects of T4 on Aquaporin 4, Astrocyte Marker GFAP and Microglial Marker Iba-1 in Frontal Cortex and Dentate Gyrus (DG) Region of Hippocampus

**Figure 3:**
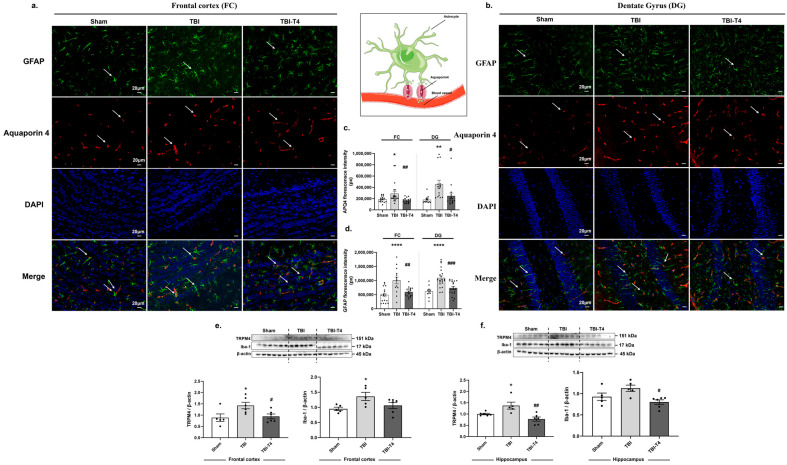
T4 maintains edema in brain by modulating aquaporin-4 levels. Dual staining of GFAP (astrocyte marker) and aquaporin-4 followed by nuclear staining (**a**) in frontal cortex; (**b**) in dentate gyrus (hippocampus); (**c**) quantification of florescence intensity of APQ4 in pixels (px). Scale bar 20 μm (**d**) quantification of fluorescent intensity of GFAP in pixels (px). Scale bar 20 μm. Protein levels of TRPM4 and Iba-1 normalized with β-actin (**e**) in frontal cortex and (**f**) in hippocampus. Data are expressed as mean ± SEM. * *p* < 0.05, ** *p* < 0.01, **** *p* < 0.0001 (sham vs. TBI). **^#^** *p* < 0.05, **^##^** *p* < 0.01, **^###^** *p* < 0.001 (TBI vs. TBI-T4). Data was analyzed using ANOVA followed by post hoc test.

Post-injury derangements lead to disruption of BBB and cerebral edema formation. To further elucidate the effect of AQP4 on astrocytes, GFAP co-expression of these markers was studied by immunofluorescence. One-way ANOVA analysis of AQP4 showed F (2, 51) = 5.62, *p* = 0.0062, in the frontal cortex and F (2, 39) = 6.83, *p* = 0.0029, in the DG region of hippocampus, followed by Sidak’s post hoc test. There was an increase in florescence intensity for AQP4 both in the frontal cortex and DG region of hippocampus in TBI group compared to sham (frontal cortex, *p* = 0.0312; hippocampus, *p* = 0.0027). However, T4 treatment reduced the fluorescence intensity of AQP4 in response to TBI (frontal cortex, *p* = 0.0057; hippocampus, *p* = 0.00163) (Figure 3a–c).

AQP4 is the major water channel of CNS, highly concentrated in the end feet of astrocytes and localized in close association with microvascular endothelial cells. It plays an important role in water movement and critically regulates neuroinflammation during injury. One-way ANOVA analysis of astrocytic marker GFAP showed F (2, 45) = 12.22, *p* < 0.0001, in the frontal cortex and F (2, 47) = 13.16, *p* < 0.0001, in the DG region of hippocampus, followed by Sidak’s post hoc test. Astrocytic marker GFAP was found to be sharply increased post-TBI in response to sham both in the frontal cortex and DG region of hippocampus (frontal cortex, *p* < 0.0001; hippocampus, *p* < 0.0001), indicating heightened inflammatory response. T4 treatment counteracted the increase observed after TBI equally in the frontal cortex and DG region of the hippocampus (frontal cortex, *p* = 0.001; hippocampus, *p* = 0.0004) (Figure 3a,b,d). We also studied TRPM4, a calcium ion channel that plays an important role in cerebral edema [22]. One-way ANOVA analysis for TRMP4 in the frontal cortex and hippocampus showed F (2, 14) = 4.673, *p* = 0.0279, and F (2, 14) = 8.958, *p* = 0.0031, respectively, followed by Sidak’s post hoc test. It was interesting to see that brain injury increased the protein levels of TRMP4 in the TBI group compared to the sham group (frontal cortex, *p*= 0.0338; hippocampus, *p* = 0.0384) while T4 treatment counteracted the effects of injury both in the frontal cortex and hippocampus (frontal cortex, *p* = 0.0466; hippocampus, *p* = 0.0017) (Figure 3e,f).

Further, we also evaluated the impact of brain injury on microglial Iba-1. One-way ANOVA analysis of Iba-1 showed F (2, 13) = 3.925, *p* = 0.0464, in the frontal cortex and F (2, 13) = 5.512, *p* = 0.0185, in the hippocampus, followed by Sidak’s post hoc test. The results showed increased levels of Iba-1 after TBI compared to sham in the frontal cortex (*p* = 0.0357) while a non-significant increasing tendency was found in the hippocampus. However, T4 had a counteracting effect in the hippocampus (*p* = 0.0112) while the frontal cortex showed no significance but possibility of reduction (Figure 3e,f).

### 2.5. Post-TBI Effect of T4 on Phenotype-Specific Astrocytes and Inflammation in Frontal Cortex and Hippocampus

**Figure 4:**
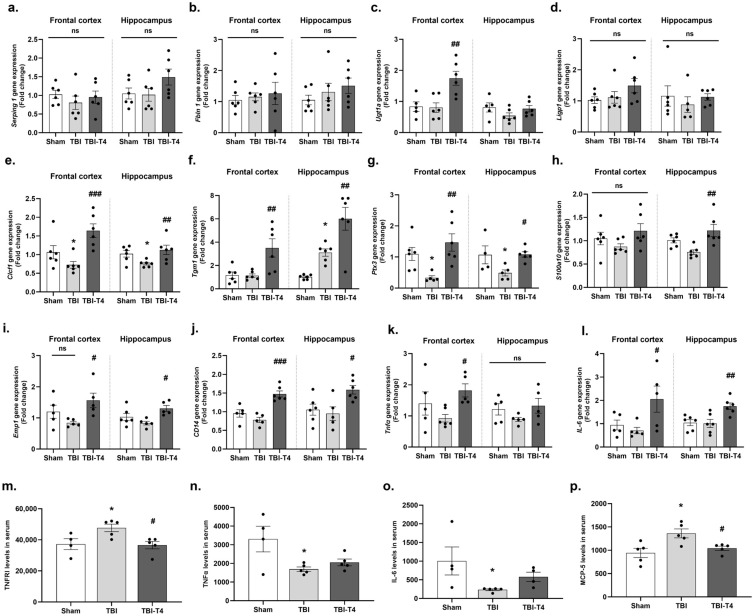
Increased expression of A2 astrocytes compared to A1 phenotype after T4 administration. Relative mRNA levels of astrocytic genes associated with A1 phenotype both in frontal cortex and hippocampus: (**a**) serping 1, (**b**) Fbln 5, (**c**) Ugt1a, (**d**) Ligp1. Relative mRNA levels of astrocytic genes associated with A2 phenotype both in frontal cortex and hippocampus: (**e**) Clcf1, (**f**) Tgm1, (**g**) Ptx3, (**h**) S100a10, (**i**) Emp1, (**j**) CD14, (**k**) Tnfα, (**l**) IL-6. Cytokine levels in serum: (**m**) TNFR1, (**n**) Tnfα, (**o**) IL-6, (**p**) MCP-5. Data are expressed as mean ± SEM, *n* = 5–6 mice/group. * *p* < 0.05 (sham vs. TBI). **^#^** *p* < 0.05, **^##^** *p* < 0.01, **^###^** *p* < 0.001 (TBI vs. TBI-T4), ns represents non-significant. Data was analyzed using ANOVA followed by post hoc test.

Based on their functions, reactive astrocytes are divided into A1 and A2 types. We found no evidence that TBI or TBI-T4 induces activation of A1-type astrocyte, i.e., *ligp1*, *serping1*, *fbln5*, and *ugt1a,* among the groups at this timepoint, probably due to the delayed timepoint in regard to A1-associated activation of inflammation (Figure 4a–d). In contrast, TBI resulted in downregulation of A2 phenotypic astrocyte-expressing neuroprotective genes (*clcf1*, *tgm1*, *ptx3*, *s100a10*, *emp1*, and *Cd14*) involved in neuronal repair, growth and survival. T4 treatment post-TBI resulted in considerable increase in mRNA levels of A2 astrocytes without affecting A1-associated astrocytic genes (Figure 4e–j). The BBB disruption allows lymphocytes, macrophages, plasma proteins to enter the brain parenchyma resulting in the activation of microglia [23] and astrocytes [24].

Next, we studied pro-inflammatory markers, TNFα and IL-6, both in the brain and in circulation. We also studied MCP-1 chemokine in circulation. One-way ANOVA analysis of *Tnfα* gene expression in the frontal cortex showed F (2, 13) = 3.851, *p* = 0.0486, followed by Sidak’s post hoc test. We did not observe significant changes in TBI vs. sham groups, while increased mRNA expression was observed with T4 treatment in TBI mice (*p* = 0.0322). Moreover, we did not observe change in hippocampus among the groups (Figure 4k). The one-way ANOVA analysis for *IL-6* mRNA expression showed F (2, 12) = 4.118, *p* = 0.0435, in the frontal cortex and F (2, 15) = 7.822, *p* = 0.0047, in hippocampus followed by Sidak’s post hoc test. We did not observe a significant difference in TBI response to sham group, while increased expression was observed with T4 treatment in TBI mice both in the frontal cortex (*p* = 0.0391) and hippocampus (*p* = 0.0064) (Figure 4l). Interestingly, circulating levels of TNFα were reduced in TBI vs. sham in consistency with a non-significant trend toward reduction that was detected in the mRNA brain-region-specific findings (one-way ANOVA: F (2, 11) = 5.135, *p* = 0.0266, followed by Sidak’s post hoc test, *p* = 0.0201) (Figure 4n). Also, one-way ANOVA analysis for serum levels of IL-6 showed F (2, 10) = 4.141, *p* = 0.0490, followed by Sidak’s post hoc test. The results showed marked reduction in TBI vs. sham group (*p* = 0.0329), while no change was observed after T4 treatment (Figure 4o). These pro-inflammatory markers have been shown to play an important role in repair mechanism at later timepoints [25]. One-way ANOVA analysis of *Tnfr1* showed F (2, 11) = 5.46, *p* = 0.0225, in serum, followed by Sidak’s post hoc test. Our result showed increased levels of *Tnfr1* in circulation 7 days post-TBI compared to sham (*p* = 0.0445). However, T4 treatment counteracted the increased levels of *Tnfr1* in response to TBI (*p* = 0.0245) (Figure 4m). TBI typically exhibits an elevation of inflammatory mediators within hours of the injury onset [26,27]. Reactive astrocytes and microglia promote the release of inflammatory cytokines and chemokines post injury resulting in BBB disruption [28]. TBI and other cerebral injuries upregulate the receptor-mediated release of tumor necrosis factor α (TNFα), which plays a crucial role on inflammation and hyperpermeability. We also measured the serum *Mcp-5* levels, and one-way ANOVA analysis showed F (2, 12) = 6.807, *p* = 0.0106. Sidak’s post hoc test revealed significant reduction in TBI vs. sham group (*p*= 0.0082), while counteracting effects were observed with T4 treatment after brain injury (*p* = 0.0389) (Figure 4p).

### 2.6. Effect of T4 on DHA and Brain Fatty Acid Binding Protein (FABP-B) After TBI

**Figure 5:**
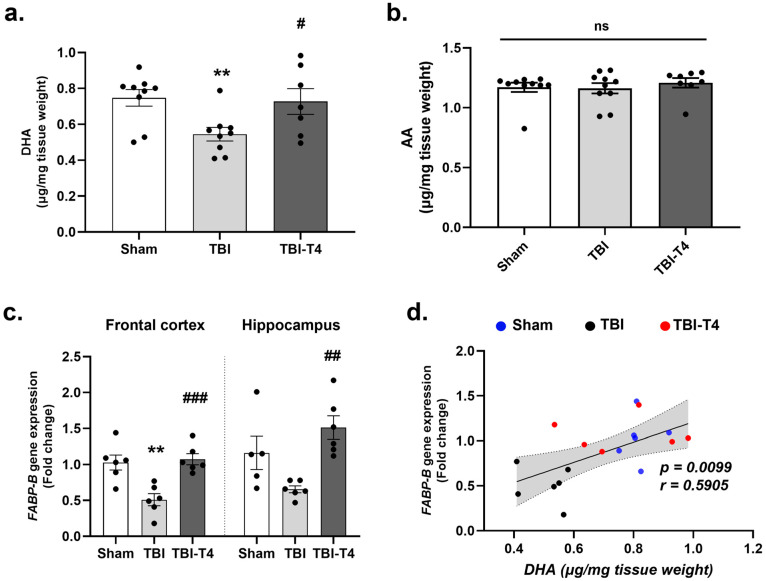
T4 modulates DHA levels and augments its transport in brain. Gas chromatography analysis in cerebral cortex: (**a**) DHA; (**b**) AA; (**c**) mRNA gene expression of FABP-B in frontal cortex and hippocampus. (**d**) Correlation between DHA (μg/mg tissue weight) and FABP5 gene expression. Data are expressed as mean ± SEM, *n* = 5–9 mice/group. ** *p* < 0.01 (sham vs. TBI). **^#^** *p* < 0.05, **^##^** *p* < 0.01, **^###^** *p* < 0.001 (TBI vs. TBI-T4), ns represents non-significant. Data was analyzed using ANOVA followed by post hoc test.

One-way ANOVA analysis of DHA showed F (2, 22) = 5.05, *p* = 0.0156, in cerebral cortex, followed by Sidak’s post hoc test. Our results showed a significant decline in brain DHA in TBI animals (μg/mg tissue weight) compared to the sham group (*p* = 0.0155), while T4 treatment normalized DHA levels (T4 vs. TBI, *p* = 0.0433) (Figure 5a). However, no significant difference was observed in arachidonic acid (AA) levels between the injury (TBI) and T4-treated groups (Figure 5b). DHA has been shown to regulate key components of brain edema, such as aquaporin-4 (AQP4), MMP-9, and the tight junction protein occludin-1 in previous studies [29,30] highlighting the positive impact of DHA on the maintenance of BBB [31]. In addition to its effects on MMP-9, DHA has the potential to reduce endothelial injury by decreasing the inflammatory response [32].

It has been shown that DHA interacts with fatty acid binding protein-brain (FABP-B) and facilitates its transport across endothelial cells lining the blood vessels [33]. One-way ANOVA analysis of *Fabp-b* showed F (2, 15) = 12.21, *p* = 0.0007, in the frontal cortex and F (2, 14) = 8.10, *p* = 0.0046, in the hippocampus, followed by Sidak’s post hoc test. There was a significant reduction in the mRNA levels of *Fabp-b* in the frontal cortex and a non-significant reducing possibility in the hippocampus 7 days post-TBI compared to the sham group (frontal cortex, *p* = 0.0019, hippocampus, *p* = 0.0794). T4 treatment in TBI mice elevated the gene expression of *Fabp-b* both in the frontal cortex and hippocampus compared to the TBI group (frontal cortex, *p* = 0.0009, hippocampus, *p* = 0.0026) (Figure 5c). Levels of DHA (μg/mg tissue weight) changed proportionally to levels of *Fabp-b* gene expression (*p* = 0.0099, r = 0.5905) (Figure 5d).

### 2.7. Effect of T4 on Mitochondrial Complex Post-TBI

**Figure 6:**
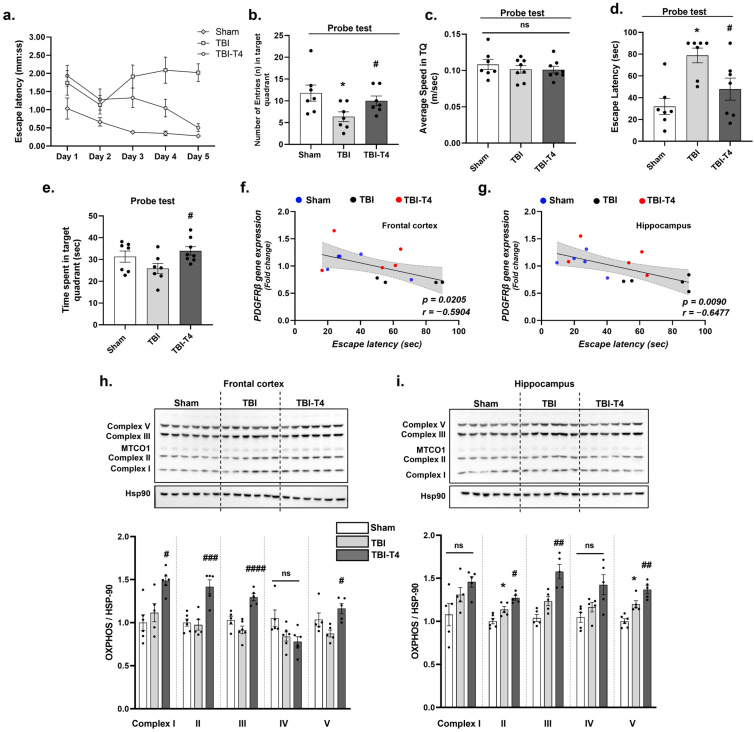
T4 ameliorates cognitive impairment. Barnes maze was used to evaluate spatial memory and exploration behavior in mice using different parameters: (**a**) escape latency during learning trials for five days; (**b**) in probe test, number of entries (*n*) in target quadrant; (**c**) average speed in target quadrant; (**d**) escape latency in probe test; (**e**) time spent in target quadrant (s); (**f**) correlation between escape latency (s) and PDGFRβ protein levels in frontal cortex; and (**g**) correlation between escape latency (s) and PDGFRβ protein levels in hippocampus. T4 altered mitochondrial OXPHOS complexes quantified using Western blot assay for all the complexes (complex I, II, III, IV, V) normalized with hsp90 (**h**) in frontal cortex, and (**i**) in hippocampus. Data are expressed as mean ± SEM, *n* = 7–8 mice/group (behavioral studies), *n* = 4–6 mice/group (OXPHOS). * *p* < 0.05 (sham vs. TBI). **^#^** *p* < 0.05, **^##^** *p* < 0.01, **^###^** *p* < 0.001, **^####^** *p* < 0.0001 (TBI vs. TBI-T4), ns represents non-significant. Data was analyzed using ANOVA followed by post hoc test.

In the frontal cortex, one-way ANOVA analysis of mitochondrial complex proteins showed the following: F (2, 14) = 9.82, *p* = 0.0022 in complex I; F (2, 13) = 15.88, *p* = 0.0003 in complex II; F (2, 13) = 19.58, *p* = 0.0001 in complex III; F (2, 14) = 4.06, *p* = 0.0405 in complex IV; F (2, 12) = 5.67, *p* = 0.0184 in complex V. This was followed by Sidak’s post hoc test. We did not find changes in mitochondrial complex protein activity in the frontal cortex of the TBI group compared to the sham group, while T4 treatment in the TBI group increased the protein level of complexes I, II, III, and V in comparison to the TBI group (T4 vs. TBI, complex I (*p* = 0.0153), complex II (*p* = 0.0005), complex III (*p* < 0.0001), complex IV (*p* = 0.7898), complex V (*p* = 0.0113)) (Figure 6h).

In the hippocampus, one-way ANOVA analysis of mitochondrial complex proteins showed the following: F (2, 12) = 3.98, *p* = 0.0470 in protein levels of complex I; F (2, 13) = 20.08, *p* = 0.0001 in complexes II; F (2, 12) = 19.77, *p* = 0.0002 in complex III; F (2, 12) = 5.57, *p* = 0.0194 in complex IV; and F (2, 13) = 25.17, *p* < 0.0001 in complex V. Sidak’s post hoc test showed an increase in the TBI group compared to the sham group (TBI vs. sham, complex II (*p* = 0.0147), complex V (*p* = 0.0044)), while T4 treatment led to a marked increased levels of complexes II, III, and V compared to the TBI group (T4 vs. TBI, complex II (0.0195), complex III (*p* = 0.0038), complex V (*p* = 0.0161)) (Figure 6i).

### 2.8. Effect of T4 on Behavioral Parameters and Cognition Post-TBI

We conducted Barnes maze behavioral testing to assess spatial memory performance. Our key findings indicate that the TBI group exhibits significant memory loss, as evidenced by an increased escape latency during the learning and probe test, number of entries in the probe test and time spent in the target quadrant in the probe test. However, T4 treatment improved memory retrieval after a learning trial for 5 days compared to the TBI group (two-way ANOVA showed F (8, 80) = 3.824, *p* = 0.0008). Tukey’s post hoc test on day 5 showed that TBI negatively affected learning behavior compared to sham (*p* = 0.0004). However, T4 treatment normalized learning after TBI (*p* = 0.0006) (Figure 6a).

In the probe test, one-way ANOVA for total entries in the target quadrant showed F (2, 18) = 3.900, *p* = 0.0392, followed by Sidak’s post hoc test. The TBI group showed fewer entries (*n*) in the target quadrant (*p* = 0.0267) and the time spent (mm:ss) in the target quadrant (*p* = 0.2130) was lower, indicating less exploratory behavior in these mice as compared to the sham group. However, T4 administration showed significant increase in the number of entries (*p* = 0.0386). One-way ANOVA for time spent in target quadrant showed F (2, 19) = 3.309, *p* = 0.050, followed by Sidak’s post hoc test. We did not observe changes in the sham vs. TBI group while T4 vs. TBI group (*p* = 0.0396) was increased (Figure 6b,e). We also observed the escape latency during the probe trial. One-way ANOVA showed F (2, 18) = 8.364, *p* = 0.0027. Sidak’s post hoc test showed a significant increase in TBI group vs. sham group (*p* = 0.0016) while T4 treatment showed marked reduction in latency (*p* = 0.0321) (Figure 6d). No change in speed was observed among the groups (one-way ANOVA, F (2, 20) = 0.4786. *p* = 0.6266) (Figure 6c). Previous studies have indicated that BBB damage is an early indicator of cognitive dysfunction in humans [34]. Our correlations indicate pericyte loss leading to cognitive decline. We observed moderate inverse proportional change between escape latency (s) and pericyte markers, *pdgfrβ* (frontal cortex, *p* = 0.0205, r = −0.5904; hippocampus, *p* = 0.0090, r = −0.6477), indicating BBB dysfunction can possibly cause cognitive impairment (Figure 6f,g).

## 3. Discussion

BBB acts as a gatekeeper to maintain CNS integrity and function. We investigated the therapeutic potential of thyroid hormone T4 to repair BBB dysfunction following TBI. We found that experimental moderate TBI disrupts BBB integrity as evidenced by loss of pericyte and endothelial cells, increased MMP-9 and TLR-4, and reduced tight junction proteins (ZO-1) and increases edema associated water channel, AQP4. TBI also had differential effects on astrocytic markers belonging to A1 (neurotoxic) and A2 (neuroprotective) categories. T4 treatment in TBI mice reduced the loss of pericytes and endothelial cells, critical regulators of vascular permeability. T4 administration also normalized levels of ZO-1, MMP-9 and TLR-4 after TBI. T4 was observed to counteract the TBI-related increase in AQP-4 associated with astrocytes, with potential mitigation of edema and inflammation. In addition, we found that TBI reduced levels of DHA in the cerebral cortex, and these effects were counteracted by T4 application. Due to the fact that DHA plays a critical role on plasma membrane function, these results emphasize the potential of T4 to preserve plasma membrane integrity and function. The promising approach of T4 in the restoration of BBB function might play a unique role in reviving the cognitive functions severely affected after brain injury.

### 3.1. Counteractive Action of T4 Treatment on BBB Integrity After TBI

Evan’s blue dye, being a relatively large molecule, is unable to cross the BBB under normal physiological condition and its extravasation provides a quantitative measure of BBB permeability [35,36]. We observed extravasation of Evan’s blue after TBI, particularly in the frontal cortex, indicative of disrupted BBB permeability; however, T4 treatment counteracted these effects of TBI. Moreover, studies have shown that increased permeability is associated with various pathological conditions, such as inflammation, infection and neurological disorders [37].

TBI reduced pericyte coverage/count in conjunction with increasing BBB permeability to Evan’s blue, and T4 treatment re-established the vascular network (endothelium, pericytes, basement membrane). Endothelial cells, along with the extracellular matrix, astrocytes and pericytes, provide a physical barrier that protects the brain from toxin entry, such that their malfunction results in various vasculature-related disorders, diabetic retinopathy and neurodegenerative disorders [38]. For example, in hypoxia, pericytes detach from perivascular locations, increasing permeability and neuronal injury [39].

This study revealed a significant loss in endothelial marker *Pdgf-b* (Platelet-Derived Growth Factor) and its receptor β (*Pdgfrβ*) on pericytes post-TBI. The exogenous administration of PDGF-B has been shown to prevent neuronal death in the hippocampus and to offer neuroprotection in cases of ischemia, highlighting its therapeutic potential [40,41]. After brain injury, PDGFRβ positive pericytes have been shown to migrate towards the injured areas to stabilize leaky blood vessels by forming scars in glial cells to seal the damaged area [42]. In addition, other endothelial and CNS-specific pericyte markers such as *Pecam* and *Atp13a5* were also reduced after TBI, most likely affecting endothelial and pericyte transcytosis, a process rapidly arrested by T4 treatment promoting BBB membrane integrity and selectivity.

### 3.2. Neuroprotective Role of T4 on Astrocytosis and Cerebral Edema

Inflammation is an important outcome following TBI, with astrocytes being the primary contributors to the release of inflammatory molecules. Disruption of BBB after brain injury or stroke results in inflammation and leukocyte recruitment to the injury site. In the current study, we focused on A1- and A2-type astrocytes where A1 phenotypic astrocytes are involved in the amplification and development of inflammation while A2 influences several neuroprotective factors that promote synaptic repair and neuronal survival after brain injury or disease [43,44,45,46]. In our study, most neuroprotective A2 astrocyte genes decreased sharply after TBI while T4 treatment counteracted these effects, emphasizing the neuroprotective action of T4. We do not intend to assign astrocytes to A1 or A2 categories but rather to assess the expression of representative genes previously reported to be enriched in reactive astrocyte states to assess the efficacy of T4 post-TBI. Astrocytes are found at the interface of blood vessels and brain parenchyma and their end feet surrounds the blood capillaries participating in the BBB [47]. Several studies have reported an increase in A1 astrocytes following TBI [48,49,50], while in the current study, neurotoxic A1 astrocyte genes were unaltered after TBI and T4 treatment. This discrepancy may be attributed to several factors, including differences in TBI models (e.g., injury severity, location, or method of induction), timepoints of analysis, brain regions examined, and species (rat) used for experimentation. It is also possible that the reactive astrocyte response in our model was more heterogeneous or leaned toward a mixed or A2-like phenotype rather than a distinct A1 profile.

TBI increased astrocytosis in conjunction with Aquaporin 4 (AQP-4) levels, while T4 counteracted these effects. AQP4 is a prominent aquaporin in the brain and also found in the kidney and striated muscle [51]. Given that AQP-4 is a recognized marker of cerebral edema [52], these results suggest an action of T4 on preventing cerebral edema and inflammatory response with subsequent preservation of BBB integrity. It has been shown that BBB disruption resulting from endothelial damage aggravates vasogenic edema and worsens prognosis, effects which are accompanied by increased AQP-4. BBB leakage after TBI is a major contributor to the inflammatory response [53], affecting water channels located by the end feet of astrocytes. Studies have shown that in hypoglycemic conditions, the end-feet pool of AQP4 changes along with degraded tight junction proteins leading to an edematous environment in the brain, neuronal injury, and inflammation [54,55,56]. Deletion of AQP4 results in restoration of BBB membrane integrity and reduction in inflammatory response in hypoglycemic patients [53]. Recent studies identified translational readthrough isoform AQP4X is highly expressed at the astrocytic end feet and regulates perivascular polarization. While canonical AQP4 is enriched in the parenchymal membrane of astrocytes. These reports highlight that changes in the total levels of AQP4 do not confirm whether changes results in vascular polarization or BBB permeability [57,58,59,60]. Moreover, differences in the injury model, severity, timepoints, animal age and sex also show variation in AQP4 polarization at the perivascular end-foot processes post-TBI [61]. Taken together, our findings reveal an overall increase in astrocytic AQP4 expression post-TBI without making inferences about isoform-specific localization. Future studies will aim to assess both total AQP4 levels and polarity in order to provide a more comprehensive picture of AQP4 regulation.

### 3.3. Impact of TBI and T4 Treatment on Vascular Rigidity

To elucidate how T4 affects vascular function, we assessed molecular indicators of hyperpermeability and tight junction proteins. We observed an increase in MMP-9 and TLR-4 in the frontal cortex and hippocampus after TBI, which is likely related to a disruption of BBB. Studies have shown that astrocytes secrete MMP-9, which increases edema and hemorrhage by affecting vascular permeability in cerebral vascular injury disease [62,63]. Thus, it is important to maintain homeostatic levels of MMP-9 to avoid hyperpermeability of toxins and injury aggravation [64]. Too much MMP-9 in the injured brain is responsible for degradation of tight junction proteins and endothelial permeability in a toll-like receptor 4 (TLR4)-dependent manner [65], with subsequent elevation of pro-inflammatory cytokines. Our current investigation showed that administration of T4 in TBI mice ameliorates hyperpermeability by normalizing levels of MMP-9/TLR4 and tight junction protein ZO-1.

### 3.4. Impact of TBI and T4 Treatment on BBB-Related Angiogenesis

In additional support for a breakdown of the BBB, we found a sharp increase in the phosphorylation of pVEGFR2/tVEGFR2 after TBI. In this study, T4 treatment resulted in the stabilization of VEGF levels after TBI that could facilitate formation of blood vessels at the site of injury, helping restore BBB integrity. VEGF is an angioneurin that plays a pivotal role in the occurrence of cerebral ischemia. Stroke, brain injury, and other pathological diseases upregulate VEGF and promote vascular leakage and brain edema [66]. TBI increases VEGF with subsequent damage to BBB integrity and aggravation of secondary brain injury [20]. Also, increased levels of VEGF and MMP-9 foster neutrophil infiltration in the basement membrane, resulting in secretion of inflammatory cytokines (IL-1β and TNFα). VEGF causes blood vessel destabilization by activation of its receptor VEGFR-2, resulting in suppression of PDGFR-β signaling by formation of VEGFR-2/PDGFR-β complex. Additionally, the interaction of VEGF and VEGFR-2 disrupts pericyte attachment to the basement membrane, resulting in membrane leakage and instability of newly formed blood vessels [67,68]. TNFα and IL-6 are early pro-inflammatory cytokines that typically peak within the first 24–72 h post-injury. In contrast, TNFR1 are persistently upregulated and shed into the circulation as a soluble form, acting as a decoy receptor to neutralize TNFα activity. The sustained increase in soluble TNFR1 may thus represent a compensatory mechanism to regulate TNFα signaling during the subacute phase of injury. TNFα and IL-6 have been known to play important roles in the repair process after injury at later timepoints [25]. Reduced levels of TNFα in the brain and serum and increased TNFR1 expression, together with elevated MMP-9 and p-VEGFR1, are consistent with the temporal dynamics of TNFα signaling. TNFα peaks early and declines later, whereas TNFR1 expression remains elevated. Importantly, we also found that MCP-5 was increased at this timepoint, indicating sustained inflammatory activity. Such persistent chemokine-driven inflammation, together with elevated TNFR1, likely contributes to the upregulation of MMP-9 and p-VEGFR1, reflecting ongoing vascular remodeling and BBB disruption in the subacute phase after TBI.

### 3.5. Influence of TBI on Thyroid Hormone Regulation

We examined the transporters, receptors, and enzymes involved in thyroid hormone (TH) metabolism, which likely play an important function in the brain. In this study, mice with TBI showed reduced expression of T4 transporters, particularly *Oatp1c1* and *Mct8*, that are involved in cretinism, a disease with severe mental illness, ataxia, and deafness [69]. However, T4 treatment restored transporters’ expression with potential effects on enabling active transportation of T4 in the brain. T4 enters the brain using two pathways, via the BBB and via the blood–CSF barrier. T4 enters endothelial cells followed by entry into the end feet of astrocytes via TH transporter OATP1C1, precisely found in the endothelial cells and choroid plexus [70].

Several clinical studies have shown reduced levels of circulation T4 depending upon the severity of injury (mild, moderate, and severe) [71]. In some cases, brain injury disrupts the hypothalamic–pituitary–thyroid axis (HPT), known as euthyroid sick syndrome with critical consequences on body functions [72]. We observed reduced thyroid hormone signaling after brain injury, leading to impairment in neuronal survival, synaptic plasticity, mitochondrial function, and inflammation response. Therefore, exogenous T4 supplementation is being explored for neuroprotection and cognitive assessment after TBI. T4 is converted to T3 with the help of *Dio2* in astrocytes. T4 treatment facilitates the efficient conversion of T4 to T3 both in the cortex and hippocampus, thus augmenting neuronal activation [73]. In the current study, *Dio2* mRNA levels were reduced in TBI mice, compromising the conversion of T4 to active T3 with subsequent effects on inflammation, metabolism, and neuronal plasticity.

### 3.6. Impact of TBI and T4 Treatment on Mitochondrial Biogenesis After BBB Disruption

TBI is associated with reductions in brain bioenergetics, and T4 is deeply involved with energy metabolism. T4 treatment in our study potentiated the assembly of mitochondrial complexes (I, II, III, V), resulting in upregulation of mitochondrial respiration. Mitochondria is the powerhouse of the cell, generating ATP via oxidative phosphorylation (OXPHOS) complexes. These complexes are known as NADH: ubiquinone oxidoreductase (complex I), succinate dehydrogenase (complex II), ubiquinol–cytochrome c oxidoreductase (complex III, or cytochrome b c1 complex), cytochrome c oxidase (complex IV), and ATP synthase (complex V). Complex I is the most critical and rate limiting enzyme involved in controlling BBB permeability, likely involving bioenergetic homeostasis [74]. Additionally, astrocytes and neurons regulate energy metabolism through the mitochondrial electron transport chain (ETC), impacting memory and neurological functions [75]. Thus, T4 treatment after brain injury might have contributed to restoring the mitochondrial biogenesis necessary for the maintenance of BBB function. A limitation of our studies is the lack of assessments of mitochondria activity, which may be necessary to detect the effects of TBI.

### 3.7. Cognitive Implications of TBI-Induced BBB Breakdown and the Therapeutic Potential of T4

It is known that cerebrovascular dysfunction exerts a heavy toll on cognition, and it is considered an early biomarker for mild cognitive impairment [76]. Our results showed a moderate association between BBB permeability and cognitive function after TBI (Figure 6f,g). Clinical studies also shown that breakdown of BBB permeability is associated with compromised cognition, as assessed by neuroimaging, neuropathological assessments, and cerebrospinal fluid biomarkers [77,78]. The present study demonstrates that traumatic brain injury (TBI) induces breakdown of the blood–brain barrier (BBB), a pathological event that compromises neuronal homeostasis and progressively impairs cognitive functions. Consistent with this, Barnes maze testing revealed marked hippocampal-dependent spatial disorientation, reflected in difficulties with both retaining familiar routes and acquiring new spatial information. The hippocampus, being central to spatial memory and learning, is vulnerable to BBB disruption, excitotoxic injury, and inflammatory response. In turn, cortical dysfunction, while not directly affected by the lesion, contributes significantly to secondary outcomes such as impairments in attention, executive control, and the adoption of effective navigational strategies. Importantly, administration of T4 showed restorative potential, mitigating hippocampal and cortical dysfunction and counteracting the cognitive deficits. These findings highlight the mechanistic link between BBB integrity, hippocampal–cortical network disruption, and impaired cognition after TBI, while emphasizing the therapeutic promise of T4 in preserving or reinstating cognitive functions.

### 3.8. Action of TBI and T4 Treatment on DHA

The current study shows a sharp reduction in omega 3 fatty acid docosahexaenoic acid (DHA) levels after TBI and suggests that targeting DHA might improve BBB integrity and cognitive functions. DHA plays an integral action on brain development, cognitive performance, and other brain functions [79], and attenuates the effects of TBI [80]. Other studies have shown the therapeutic potential of DHA in reducing brain edema, AQP4-related hyperpermeability marker MMP-9 [29,30], brain inflammation [81] and inflammatory cell infiltration [82,83]. We also assessed the transcriptomic levels of DHA transporter *Fabp-b*, which is known to facilitate the diffusion of substances/fatty acids across the endothelial cells lining the blood vessels [84]. Our results showed that T4 treatment reinstated normal DHA levels together with increasing *Fabp-b* transporter that could contribute to relieve the impaired BBB permeability in mice with TBI. FABP-B is exclusively found in the brain and promotes transcellular permeability resulting in improved neurological function by ameliorating BBB dysfunction [85,86]. Hence, deficiency in FABP-B causes inefficient transport of DHA in the brain, resulting in neurological and cognitive defects [87].

### 3.9. Translational Relevance and Clinical Implications of T4 Therapy in TBI

Studies revealing that BBB breakdown causes hippocampal–frontal cortex dysfunction aligns with clinical data showing persistent microvascular leakage and neuroinflammation in patients with mild-to-severe TBI. These studies portray BBB integrity as a therapeutic target to treat cognition [88,89]. Because levothyroxine (T4) is FDA-approved, affordable, and widely available, repurposing T4 might be suitable for mitigating BBB-linked cognitive deficits. T4 seems to regulate endothelial tight junctions, to reduce microglial activation, and to normalize vascular integrity, resulting in improved hippocampal-dependent spatial memory and frontal executive control. Clinical practice might face challenges with regard to dose, route and treatment length of T4, previous thyroid level status, and variability in injury severity and lesion location that influence treatment efficacy.

### 3.10. Limitations of Using T4

While exogenous T4 treatment has shown promise in mitigating cerebral edema and promoting neuroprotection, it carries several important limitations. Inappropriate T4 dose selection can induce systemic hyperthyroidism, leading to metabolic disturbances, weight loss, and cardiovascular stress that may lead to neurological outcomes. Moreover, T4 might disrupt the hypothalamic–pituitary–thyroid (HPT) axis through negative feedback, suppressing endogenous hormone production and potentially altering long-term neuroendocrine balance. Its broad systemic activity affects multiple organs beyond the brain, making it difficult to isolate CNS-specific effects. Additionally, the therapeutic window for T4 is narrow, requiring precise dosing and timing to avoid toxicity or inefficacy. These factors underscore the need for cautious interpretation and rigorous control when evaluating T4’s therapeutic potential in injury models. The study assessed spatial learning and memory deficits using the Barnes maze, but motor function was not directly evaluated. However, as no differences in velocity were observed among groups during the task, it is unlikely that motor impairments confounded the cognitive outcomes.

## 4. Material and Methods

### 4.1. Animal Studies

Nine-week-old male C57BL/6J mice (Jackson Laboratory, Bar Harbor, ME, USA) weighing 20–25 g were used. The mice colony was maintained under standard housing conditions at controlled temperature of 22–24 °C with free access to food and water ad libitum. A 12 h day/light cycle was maintained. For experiments, mice were randomly assigned to sham, TBI (injury), and T4 treatment groups. Mice were given acute doses of T4 intraperitoneally after TBI. The mice were then sacrificed, and tissues were collected and flash-frozen, followed by storage in −70 °C until further use.

### 4.2. Fluid Percussion Injury

FPI is a well-established model that closely mimics the diffuse brain injury and pathophysiology observed in many human TBI cases, including blood–brain barrier disruption, cerebral edema, and cognitive deficits [90]. The mice received two intraperitoneal injections (acute treatment) of T4, first injection at 1 h and a second injection at 6 h post-TBI at the dose of 1.2 μg/100 g body weight with free access to food and water ad libitum [91,92]. Mice were subjected to fluid percussion injury (FPI) as previously described [93]. Briefly, mice were anesthetized with isoflurane (2.5% in 100% O_2_) through a nose cone, using the anesthesia system (Vet Equip Inc., Marsing, ID, USA). After the surgical site was shaved, the head was fixed in a stereotaxic apparatus and eye ointment was applied for corneal protection. The surgical site was then cleaned using sterilized swabs of povidone iodide and 70% ethanol alternatively. A midline incision was made over the skull, and the skin and fascia were retracted. Using a high-speed drill (Dremel, WI, USA), craniectomy was performed (centered at the 3 mm posterior relative to bregma and 6 mm lateral to the midline). A rigid plastic injury cap made from a rigid Luer-lock needle hub was placed over the craniectomy site with the help of dental acrylic cement. Once the dental cement has hardened, the anesthesia was discontinued. The hub was filled with non-pyrogenic saline to avoid drying in the exposed area of brain, and the hub was then attached to the FPI device. At the first sign of hind-limb withdrawal to a paw pinch, a moderate pulse of saline (approximately 1.5 atm) was imposed on the dura by releasing the pendulum onto the fluid-filled cylinder. We employed a moderate level of injury with a pulse pressure range of 1.8–2.1 atm and righting reflex of 7–10 min. After the injury, the mice were allowed to wake up shortly. Wake-up time was recorded for each mouse. Once the heartbeat was normalized and the mouse was awake, it was subjected to re-anesthetization, hub removal, and skin suture. Triple antibiotics were applied on the sutures, and the mice were placed on the heating pad in a recovery chamber to be fully active before being returned to their cages. Carprofen (pain killer) was injected subcutaneously in mice 45 min pre-TBI and 24 h post-TBI at the dose of 5 mg/kg for 3 days. Sham mice underwent craniectomy but without injury. The mortality rate was less than 1% and often resulted in 100% survival, consistent with other studies [94]. TBI-group mice received intraperitoneal injections of PBS as a vehicle control corresponding to the TBI-T4 treatment group. We aimed to assess the therapeutic potential of T4 in ameliorating diffuse injury-induced impairments, particularly blood–brain barrier dysfunction and spatial learning deficits, both of which are robustly represented in the FPI model.

### 4.3. Barnes Maze

Barnes maze testing was performed to access the learning and memory functions of the mice. The maze is circular dry land made from acrylic plastic to form a disk 1 cm thick and 122 cm in diameter, containing 20 equally spaced holes of diameter 5 cm each. The apparatus is connected to an overhead camera, and the platform is surrounded by three distal cues surrounding the disk, brightly illuminated by four overhead halogen lamps to provide aversive stimulus. The apparatus is connected to the computer with a camera installed on it to visualize and record the movement of the mice. Briefly, the test is divided into habituation (day 1), learning (day 2–day 6) and probe test (day 7). The Barnes maze test was conducted 24 h post-TBI. On day 1, the mice were habituated to the maze by leaving them on the disk for 2 min (without escape box), in order to become accustomed to the room, table, and maze layout. A trial was started by placing the animal in the center of the maze covered under a cylindrical box (transparent for habituation, translucent for learning, and opaque for probe test). After a pause of 10 s, the box was lifted and the test begins. For learning, the mice were given 120 s (maximum time) to find the escape hole, and the test was terminated as soon as the mice entered the target hole (two trials/mouse with intertrial interval of 20–30 min). The mice that do not find the escape box are gently guided to it by holding their tails, helping them to locate the target hole/escape box. On day 7, the escape box was removed, and the maze was divided into four quadrants (A, B, C, D) and the quadrant with the target hole is determined as the target quadrant. The target hole is located in the middle of the target quadrant. The probe test was conducted for 90 s (two trial per mouse), in order to evaluate their memory retention. Parameters such as escape latency during learning, escape latency during probe test (minimum time to approach the target hole), time spent (s) and number of entries (*n*) in the target quadrant were analyzed.

### 4.4. Evan’s Blue

Mice were weighed to measure the amount of dye that needs to be injected in the mice. Two percentage of Evan’s blue solution in saline was used to study the dye extravasation. The dye was injected intraperitoneally (~300 μL), two hours before sacrificing the mice. The hands and feet of the mice turn blue, indicating effective circulation of dye.

For immunofluorescence, the mice were perfused to remove the blood with 1× PBS and the brain was isolated. If blood is present, it gives autofluorescence and can interfere with Evan’s blue fluorescence intensity. Immediately after brain isolation, blocks were prepared using OCT. Further, region-specific fresh-frozen brain sectioning was performed, particularly focusing on the frontal cortex and hippocampus (section thickness—8 micron). After sectioning, slides were directly observed under a fluorescent microscope ~595 nm.

For quantitative analysis, the isolated brain region, frontal cortex and hippocampus were homogenized using a tissue grinder containing 500 μL of 50% trichloroacetic acid. The homogenate was centrifuged for 20 min at 10,000× *g* and the supernatant diluted 4X in ethanol. A standard curve was produced using different concentrations of Evan’s blue in ethanol, i.e., 1000, 100, 10, 1, 0.1, 0.01 μg/mL. Finally, for recording the fluorescence, 30 μL of supernatant and 90 μL of 95% ethanol were mixed. The fluorescence was recorded at the excitation of 620 nm and emission at 680 nm using plate reader instrument (Tecan Spark, Morgan Hills, CA, USA).

### 4.5. Immunoblotting

Brain homogenates from the frontal cortex and hippocampus were prepared using RIPA lysis buffer (ThermoFisher Scientific, USA) with phosphatase inhibitor cocktail. Lysates were homogenized using hand homogenizer and centrifuged at 13,000 rpm for 30 min and the supernatant was collected. The total protein was then measured using BCA method-based protein estimation kit (Pierce, ThermoFisher Scientific, Waltham, MA, USA), and 30 μg protein was loaded on gel to perform PAGE and Western blotting. The percentage of gel to be used was selected based on the molecular weight of the desired protein. Further, the membrane was blocked using 5% BSA in TBST. Next, the membrane was washed with 1× TBST 3 times, followed by overnight incubation of primary antibody at 4 °C. Bound antibody was visualized using HRP conjugated secondary antibody (1 h at room temperature), and the membrane was developed using a gel documentation instrument (Bio-Rad, Hercules, CA, USA). The antibodies used to identify changes in BBB integrity and permeability with brain injury and counteractive action of T4 are mentioned in Table 1.

### 4.6. Real-Time PCR

RNA was isolated from the brain (frontal cortex and hippocampus) using total RNA kit II (Omega BIO-TEK, Norcross, GA, USA). First-strand cDNA was synthesized from 1 μg RNA using c-DNA synthesis kit (Applied Biosystems, Waltham, MA, USA). Real-time PCR was performed from 10 ng cDNA. Various BBB-related genes were studied using gene-specific primers as mentioned in Table 2. As previously described, CFX-96 (Bio-Rad, USA) instruments were used to run the reaction using SYBR Green (Applied biosystems, USA). The fold change was calculated using 2^−ΔΔCt^ method. The blood–brain barrier-related primers were designed for studying gene expression in the brain. In this study, GAPDH was used as the housekeeping gene for normalization because it is widely reported and commonly employed in studies of TBI [95]. To ensure the reliability of GAPDH amplification, we confirmed primer efficiency using 18S rRNA. While 18S was not used for normalization, this step validated that GAPDH primers performed efficiently. In the FPI model, we observed consistency in GAPDH performance.

### 4.7. Gas Chromatography: Fatty Acid Analysis

DHA and AA brain levels were studied using gas chromatography. Total lipids were extracted from the cerebral cortex of brain tissue according to the method of Bligh & Dyer (1959). Frozen brain regions were homogenized in chloroform/methanol (2:1 *v*/*v*), containing 50 μg/mL of butylated hydroxytoluene to prevent lipid oxidation during lipid isolation. Tricosanoic acid methyl ester (C23:0) and DHA were used as an internal standard. For lipid isolation, tissues were homogenized using motorized homogenizer and subjected to the extraction of total lipids. Fatty acid methylation was performed by heating at 90 °C for 1 h with boron trifluoride–methanol reagent (14% *w*/*v*). Extracted lipids were analyzed using Clarus 500 gas chromatograph (GC; PerkinElmer, Shelton, CT, USA) with auto sampling function. An Elite-WAX column (60 m, 0.32 mm internal diameter, PerkinElmer) was used with hydrogen as the carrier gas. GC oven temperature was initially held at 140 °C for 2 min and raised with a gradient of 5 °C/min until 250 °C and held for 10 min. The injector and detector were maintained at 250 °C and 300 °C, respectively. The total run time for each sample was 34 min. Fatty acids were identified and quantified by comparison with standard peak (Supelco 37-component FAME Mix, Sigma-Aldrich, St. Louis, MO, USA) and GLC reference standard 682 (Nu Check Prep, Inc., Elysian, MN, USA).

### 4.8. Immunofluorescence for AQP4 and GFAP

The functional, morphological alterations of BBB were assessed by AQP4 and GFAP (astrocyte) immunostaining. After isolating the brain and freezing them in OCT, the sections were made in cryostat (RWD life science, Shenzhen, China). Based on the bregma, brain regions showing the frontal cortex were collected followed by serial sectioning of the hippocampus. These fresh-frozen sections were then collected and kept in −80 °C. For staining, the slides were kept at room temperature for 10 min before starting the staining procedure. The sections were fixed with 4% PFA for 7 min at 37 °C. Further, slides were washed with 1× PBS three times for 10 min each followed by permeabilization with 0.3% triton-X-100 for 10 times with 1× PBS for 10 min each. After blocking, slides were washed once with 1× PBS for 10 min followed by primary antibody incubation for 1 h at room temperature or overnight at 4 °C. Lastly, the slides were washed with 1× PBS three times for 10 min each and then incubated with secondary antibody (Alexa fluor) for 1 h at room temperature. For co-staining, the two primary antibodies are added one after the other and must be accompanied with washing steps as mentioned above. However, for secondary antibody incubation, the two antibodies are incubated together. The slides were mounted using DAPI with mounting media. The slides were then observed under a fluorescent microscope.

### 4.9. Mouse Cytokine Array

The serum markers TNFα, IL-6, MCP-5 and TNFR1 were performed using mouse cytokine array (Abcam, Cambridge, MA, USA; catalog no. ab133993) as per the instructions provided in the manual.

### 4.10. Statistical Analysis

All statistical analyses were performed in Graph Pad Prism 9 (version 9.3.0, San Diego, CA, USA). All results are represented as mean ± standard error. All other datasets were analyzed using one-way analysis of variance (ANOVA) followed by Holm–Sidak post hoc test for multiple comparison to determine significant differences among groups. The groups considered for multiple comparisons were sham vs. TBI and TBI vs. TBI-T4. A value of *p* < 0.05 was considered as statistically significant. Multiple comparisons were not performed for datasets where the ANOVA results were not statistically significant. Two-way ANOVA was used to analyze escape latency during learning, followed by Tukey’s test.

## 5. Conclusions

**Figure 7:**
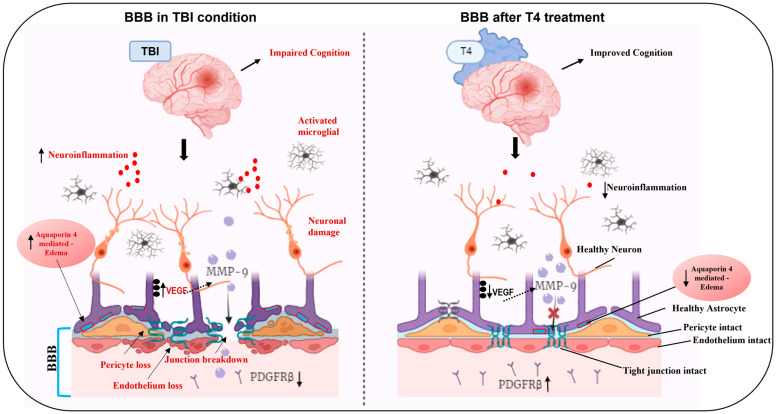
Graphical abstract: In traumatic brain injury (TBI), the neurovascular unit forming the blood–brain barrier (BBB) is compromised, disrupting the regulated exchange of substances across the membrane. This disruption damages pericytes and endothelial cells while breaking down the tight junction protein ZO-1. Additionally, the protein levels of the key hyperpermeability inducers MMP-9 and TLR-4 are significantly altered. Aquaporin-4 associated with astrocytes is markedly increased, contributing to edema and neuroinflammation after TBI. Notably, T4 administration reversed the outcomes from TBI and stabilizes the BBB. T4 regained the pericyte and endothelium loss and repaired the tight junction protein, ZO-1. T4 enabled stabilization of neuroinflammation and edema formation by reducing aquaporin-4. Overall, T4 facilitated repair of BBB leading to improved cognitive functions. Created with BioRender.com (content partially generated using the free version 4.0).

In conclusion, our study underscores the potential of T4 in facilitating brain injury recovery by restoring pericytes and endothelial cells, crucial for maintaining vascular integrity. T4 stabilizes the tight junction protein ZO-1, mitigates hyperpermeability and edema formation, and suppresses AQP4 and MMP-9/TLR-4 protein levels in critical brain regions such as the frontal cortex (linked with emotions) [96] and hippocampus (related to cognition and learning) [97]. Moreover, T4 enhances mitochondrial biogenesis and restores DHA levels, alleviating vascular leakage and inflammation, ultimately leading to improved neurological function and cognitive abilities. Furthermore, our data indicate that some effects are more pronounced in the frontal cortex, while others are more evident in the hippocampus, with T4 administration exerting beneficial effects in both regions. The study reflects an integrated hippocampal–frontal cortex network responsible for BBB breakdown. Within this framework, T4 improved spatial learning/memory, supporting a network-level (i.e., non-region-restricted) benefit that we interpret as consistent with BBB stabilization and downstream circuit recovery. Accordingly, overall, T4 seems to act by enhancing cognition through hippocampal–frontal cortex interactions (Figure 7).

## Figures and Tables

**Table 1:** List of antibodies.

Antibody	Company	Catalog No.	Molecular Weight (kDa)
MMP-9	Cell signaling (Danvers, MA, USA)	13667	92
TLR-4	Santa Cruz (Dallas, TX, USA)	sc-293072	95
pVEGFR2	Cell signaling	3770	230
VEGFR2	Cell signaling	9698	210 and 230
ZO-1	Cell signaling	8193	220
OXPHOS	Invitrogen (Carlsbad, CA, USA)	45-8099	55, 48, 40, 30, and 20
Iba-1	Fujifilm Wako (Minato, TYO, Japan)	019-19741	17
TRMP4	Novus bio (Centennial, CO, USA)	NBP3-07990	151
β-actin	Santa Cruz	sc-47778	45

**Table 2:** List of mouse primer sequences used in quantitative real-time PCR.

Gene	Accession Number	Forward Primer (5′-3′)	Reverse Primer (5′-3′)
*PDGF*	NM_001411620.1	AAGTGTGAGACAATAGTGACCCC	CATGGGTGTGCTTAAACTTTCG
*PDGFRβ*	NM_008809.2	CAAGAAGCGGCCATGAATCAG	CGGCCCTAGTGAGTTGTTGT
*PECAM*	NM_001032378	ACGCTGGTGCTCTATGCAAG	TCAGTTGCTGCCCATTCATCA
*ATP13a5*	NM_012675.3	GAGGTGTTTGGCTACCATACC	GGGATGCAACTGGTCCACA
*GLUT 1*	NM_011400.3	GCAGTTCGGCTATAACACTGG	GCGGTGGTTCCATGTTTGATTG
*OATP1C1*	NM_021471	GGGCCATCCTTTACAGTCGG	CCTTCTCTCTATCTGAGTCACGG
*MCT8*	NM_009197	CGGCTGGATAGTGGTGTTTG	TGGAGTAGAGGATACCAACAGAG
*Dio2*	NM_010050	CAGCTTCCTCCTAGATGCCTA	CTGATTCAGGATTGGAGACGTG
*THα*	NM_178060	TTTCGCCGCACAATCCAGAA	GGTGATCTTGTCGATGACACAG
*THβ*	NM_001113417	GGACAAGCACCCATCGTGAAT	CTCTGGTAATTGCTGGTGTGAT
*FABP-B*	NM_010634	AAAGAGCTAGGAGTAGGACTGG	TGTTGCCATCACACGTAATGA
*Serping1*	NM_009776.3	ACAGCCCCCTCTGAATTCTT	GGATGCTCTCCAAGTTGCTC
*Fbln5*	NM_001413785.1	CTTCAGATGCAAGCAACAA	AGGCAGTGTCAGAGGCCTTA
*Ugt1a*	NM_201645.2	CCTATGGGTCACTTGCCACT	AAAACCATGTTGGGCATGAT
*Ligp1*	NM_021792.5	GGGGCAATAGCTCATTGGTA	ACCTCGAAGACATCCCCTTT
*Clcf1*	NM_019952.6	CTTCAATCCTCCTCGACTGG	TACGTCGGAGTTCAGCTGTG
*Tgm1*	NM_001161714.1	CTGTTGGTCCCGTCCCAAA	GGACCTTCCATTGTGCCTGG
*Ptx3*	NM_008987.3	AACAAGCTCTGTTGCCCATT	TCCCAAATGGAACATTGGAT
*S100a10*	NM_009112.2	CCTCTGGCTGTGGACAAAAT	CTGCTCACAAGAAGCAGTGG
*Emp1*	NM_001288628.1	GAGACACTGGCCAGAAAAGC	TAAAAGGCAAGGGAATGCAC
*CD14*	NM_009841.4	GGACTGATCTCAGCCCTCTG	GCTTCAGCCCAGTGAAAGAC
*Tnfα*	NM 001278601.1	CAGGCGGTGCCTATGTCTC	CGATCACCCCGAAGTTCAGTAG
*IL-6*	NM_001314054.1	TGAACAACGATGATGCACTTG	CTGAAGGACTCTGGCTTTGTC
*GAPDH*	NM_017008.4	GGGCTCTCTGCTCCTCCCTGT	ACGGCCAAATCCGTTCACACC

## Data Availability

The original contributions presented in this study are included within the article. Further enquiries can be directed to the corresponding author.
